# Complete Blood Count-Derived Inflammatory Markers Changes in Dogs with Chronic Inflammatory Enteropathy Treated with Adipose-Derived Mesenchymal Stem Cells

**DOI:** 10.3390/ani12202798

**Published:** 2022-10-17

**Authors:** José Ignacio Cristóbal, Francisco Javier Duque, Jesús Usón-Casaús, Rafael Barrera, Esther López, Eva María Pérez-Merino

**Affiliations:** 1 Department of Animal Medicine, Veterinary Faculty, University of Extremadura, 10003 Caceres, Spain; 2 Stem Cell Therapy Unit, Jesús Usón Minimally Invasive Surgery Centre, 10004 Caceres, Spain

**Keywords:** cell therapy, chronic inflammatory enteropathy, neutrophil-to-lymphocyte ratio, platelet-to-lymphocyte ratio, systemic immune-inflammation index

## Abstract

**Simple Summary:**

Mesenchymal stem cells exhibit anti-inflammatory properties, and their administration to dogs with chronic inflammatory enteropathy has been shown to be safe and effective. Neutrophil/lymphocyte ratio, platelet/lymphocyte ratio, and the systemic immuno-inflammation index are considered novel biomarkers to assess the inflammatory status of patients. The present study aimed to compare the clinical evolution and the changes in these inflammatory biomarkers in dogs with chronic enteropathy before and after cell therapy. The values of the three inflammatory biomarkers were higher in dogs with chronic enteropathy before the treatment compared to the values obtained from healthy dogs. After treatment, those values decreased significantly over time, and nine months later, no difference was observed between healthy dogs and dogs with chronic enteropathy in two of the three studied markers. A relationship between the amelioration of the clinical signs and the decrease in the blood inflammatory markers was also established. These results demonstrate that the clinical improvement of patients with chronic enteropathy treated with stem cells is accompanied by a normalization of the inflammatory biomarkers studied.

**Abstract:**

Adipose-derived mesenchymal stem cells (Ad-MSCs) exhibit anti-inflammatory and immunomodulatory activities. The neutrophil-to-lymphocyte ratio (NLR), platelet-to-lymphocyte ratio (PLR), and systemic immune-inflammation index (SII) have been reported as novel biomarkers of the inflammatory state; however, they have never been examined in dogs with chronic inflammatory enteropathy (CIE) treated with Ad-MSCs. This study aimed to compare the clinical evolution and the changes in the NLR, PLR, and SII in dogs with CIE before and after cell therapy. Sixteen dogs with CIE were administered a single intravenous dose of Ad-MSCs. The canine chronic enteropathy clinical activity index (CCECAI), NLR, PLR, and SII were assessed before treatment (T0) and at 2 (T2) and 9 (T9) months post-treatment and compared over time and with the reference values obtained from a group of healthy dogs. NLR, PLR, and SII were significantly increased at T0 compared to the reference values, decreasing significantly over time. At T9, the NLR and SII did not differ from the reference values, but PLR remained above the reference values. A correlation was observed between CCECAI and the three markers. These findings show that the clinical improvement of dogs with CIE treated with Ad-MSCs is accompanied by a normalization of the inflammatory status.

## 1. Introduction

Chronic inflammatory enteropathy (CIE) describes a group of disorders of the canine enteral tract characterized by persistent gastrointestinal signs whose diagnosis is based on histologically confirmed inflammation in the intestinal mucosa after excluding other identifiable digestive and extra-digestive causes of vomiting and diarrhea [1].

Based on the patient’s response to treatment, the enteropathy can be considered food-responsive (FRE), antibiotic-responsive (ARE), immunosuppressant-responsive (IRE), or nonresponsive (NRE) [2]. The terms IBD and IRE are considered synonymous for dogs in which the histology has confirmed the presence of intestinal inflammation. Canine IRE is comparable to Crohn’s disease (CD) in humans [1].

Corticosteroids and other immunosuppressants are commonly used to treat CIE; however, nonresponsiveness and adverse reactions limit their usage [3,4,5]. Adipose-derived mesenchymal stem cells (Ad-MSCs) are a promising alternative therapy to treat chronic enteropathies in dogs and cats, which has been shown to be safe and effective [2,6,7,8]. After administration, Ad-MSCs not only migrate directly to the site of inflammation but also exert indirect anti-inflammatory effects through secretory factors [9,10,11,12].

Moreover, novel biomarkers related to the inflammatory status of patients have been developed [13]. The neutrophil-to-lymphocyte ratio (NLR), platelet-to-lymphocyte ratio (PLR), and systemic immune-inflammation index (SII) have been considered new markers for the assessment of the severity of ulcerative colitis in humans [14,15,16]. In dogs, the NLR and PLR also provide additional information regarding the severity of CIE [17,18,19]. However, the SII has not been investigated in veterinary patients.

Despite the immunomodulatory and anti-inflammatory properties of the MSCs [11,12,20] and the usefulness of NLR, PLR, and SII as indices of systemic inflammation [17,21,22], these markers have never been examined in patients treated with Ad-MSCs.

Therefore, the present study aimed to compare the clinical evolution and the changes in the NLR, PLR, and SII in dogs with CIE before and after cell therapy.

## 2. Materials and Methods

### 2.1. Animals

The study included dogs diagnosed with CIE at the Internal Medicine Unit of the Veterinary Teaching Hospital of the UEx (VTH-UEx). The Animal Care and Use Committee of the UEX and the Regional Government approved the study’s design, and all the clients signed an informed consent form.

The diagnosis of CIE was confirmed by excluding other causes of chronic diarrhea based on routine diagnostic tests. All the referred patients were examined again at the VTH-UEx, and the tests were repeated. The diagnostic procedure included a complete blood count, chemistry profile, urinalysis, serum trypsin-like immunoreactivity test, serum canine pancreatic lipase immunoreactivity test, serum cobalamin and folate concentrations, stool examination, abdominal radiography, and ultrasound examination, as well as a histopathological review of mucosal biopsy specimens obtained via both gastroduodenoscopy and ileocolonoscopy. Gastrointestinal biopsies were assessed according to the criteria proposed by the World Small Animal Veterinary Association Gastrointestinal Standardization Group for diagnosing gastrointestinal inflammation in dogs and cats [23,24]. None of the dogs underwent immunosuppressant therapy two weeks prior to endoscopy.

The CCECAI [25] is a standard index used to evaluate the severity of the disease in the dogs of this study by assessing attitude and activity, appetite, vomiting, stool consistency, stool frequency, weight loss, serum albumin concentration, and presence/absence of ascites and pruritus and scored from 0 to 3. After adding the scores from each item, a total score is obtained, according to which the disease will be considered insignificant, 0–3; mild, 4–5; moderate, 6–8; severe, 9–11; and very severe, ≥12.

Food (with hydrolyzed or novel protein diets) and antibiotic (with metronidazole or tylosin) trials were completed before administering immunosuppressive drugs. The diet and antibiotic trials were repeated at the VTH-XXX in the referred dogs. Immunomodulatory therapy was administered using prednisolone, budesonide, cyclosporine, or chlorambucil, either alone or in combinations at the clinician’s discretion.

The criteria for patient inclusion were adult dogs (≥1 year of age) with histopathologic confirmation of inflammation in intestinal biopsies and an inadequate response to immunosuppressants, defined as a reduction of <30% in the CCECAI scores after immunosuppressive therapy compared with their corresponding pretreatment values. Patients with pregnancy, sepsis, extreme physical impairment, and concomitant diseases were excluded.

A group of healthy dogs that were routinely examined at the VTH-XXX served to establish the reference values for each study parameter. The inclusion criteria for this group were healthy adult dogs (>1 year) whose clinical history, physical examination, blood hematology and biochemistry, urinalysis, and fecal examination did not reveal any abnormality. Dogs with digestive signs or signs of any other transient disease that were reported during the last month were excluded.

### 2.2. Isolation and In Vitro Expansion of Adipose-Derived Mesenchymal Stem Cells (Ad-MSCs)

Ad-MSCs were isolated from subcutaneous adipose tissue in a conventional surgery of female dog castration, as previously described [8]. Briefly, adipose tissue was digested using collagenase type V, washed, and filtered. The cells obtained were cultured and expanded in Dulbecco’s Modified Eagle Medium (DMEM) culture medium with 10% fetal bovine serum (FBS) and penicillin/streptomycin at 37 °C and 5% CO_2_. Adherent cells were phenotypically characterized by flow cytometry following the specifications of the International Society for Cellular Therapy guidelines [26] and in vitro differentiated towards osteogenic, adipogenic, and chondrogenic lineages. Expanded cells were cryopreserved until the day of administration, when the cells were thawed and resuspended in 50 mL of physiological saline solution. All the procedures described above were performed at the Minimally Invasive Surgery Centre-Unit 14 from Nanbiosis (https://www.nanbiosis.es, accessed on 5 February 2022).

### 2.3. Study Protocol

A 3-week washout period for immunosuppressive drugs was mandated before the administration of MSCs. Dogs that fulfilled the inclusion criteria received a single dose of Ad-MSCs. The thawed cells were resuspended and diluted in physiological saline to a final volume of 100–250 mL (according to animal weight). The infusion was administered over 30 min through a peripheral intravenous cannula at a target dose of 4 × 106 cells/kg body weight. The dogs were monitored during the infusion and for 60 min prior to discharge.

A physical examination was performed, and blood samples were collected just before the treatment (T0) and during subsequent visits at 2 and 9 months (T2 and T9) after treatment.

Whole blood was used for routine hematology with differential white blood cell counts, which was performed at the Clinical Pathology Laboratory of VTH-XXX using an automatic analyzer (Spincell 5 Compact, Spinreact^®^, Barcelona, Spain). An automatic leukocyte count was performed to obtain the absolute value of each white blood cell type. Serum chemistry profile was performed using a Saturno 100 VetCrony^®^ automatic analyzer (Crony Instruments, Rome, Italy). White blood cell, absolute platelet, neutrophilic, and lymphocytic counts were recorded, and the CCECAI, NLR, PLR, and SII were calculated for each time point. The NLR and PLR were calculated from the differential count by dividing the absolute neutrophil and platelet counts, respectively, by the absolute lymphocyte count. The SII values were calculated using the formula: platelet × neutrophil/lymphocyte counts.

A single blood sample was collected from each recruited healthy dog to obtain the normal values for neutrophil, platelet, and lymphocyte counts, NLR, PLR, and SII. The differences between these reference values and those obtained from the dogs with CIE at each time point (T0, T2, and T9) were analyzed.

### 2.4. Statistical Study

Data were analyzed using SPSS v22.0 (IBM Corp., Armonk, NY, USA). The Shapiro–Wilk test was used to determine the normality of the quantitative variables. Since most of the data were not normally distributed, their descriptive statistics were reported in terms of median and range (min-max).

Changes over time in the neutrophil, lymphocyte, and platelet counts, and NLR, PLR, and SII values in the treated dogs were compared using the Friedman rank test with post hoc Wilcoxon comparisons. The Mann–Whitney U test was used to compare the values of the treated dogs with their respective reference values. The effect size was calculated using Cohen’s d statistic when significant differences were detected.

Because the values were not normally distributed, correlation analysis between the inflammatory ratios and the CCECAI was carried out using Spearman’s rho. Statistical significance was set at *p* < 0.05.

## 3. Results

### 3.1. Population

A total of 16 dogs with CIE were enrolled in this study: three Yorkshire Terrier, two mixed breeds, and one each of Boxer, American Staffordshire Terrier, Bichon Maltese, American Pitbull Terrier, French Bulldog, Pomeranian, Spanish Mastiff, Spanish Water Dog, Greyhound, German Shepherd, and Poodle. There were 11 males (two neutered) and five females (two spayed). The median age and body weight was 4.3 years (range, 1–14 years) and 13.7 kg (range, 3–41 kg).

Histological evaluation showed the presence of lymphocytic-plasmacytic inflammatory infiltrates in all the dogs.

The reference value for each parameter was obtained from a group of 20 healthy dogs comprising 11 males and 9 females. Their ages ranged from 1 to 9 years (median, 3.6 years). The breeds included five mixed breeds, five Labrador Retrievers, three Border Collies, two Patterdale Terriers, and one each of Dalmatian, English Setter, German Shepherd, Spanish Mastiff, and Jack Russel Terrier. Age and sex did not differ between the healthy and treated dogs.

The descriptive statistics of the clinical indices, absolute neutrophil counts, lymphocyte, and platelet counts, NLR, PLR, and SII from the dogs with CIE and the reference values are reported in Table 1.

### 3.2. Clinical Indices

**Figure 1:**
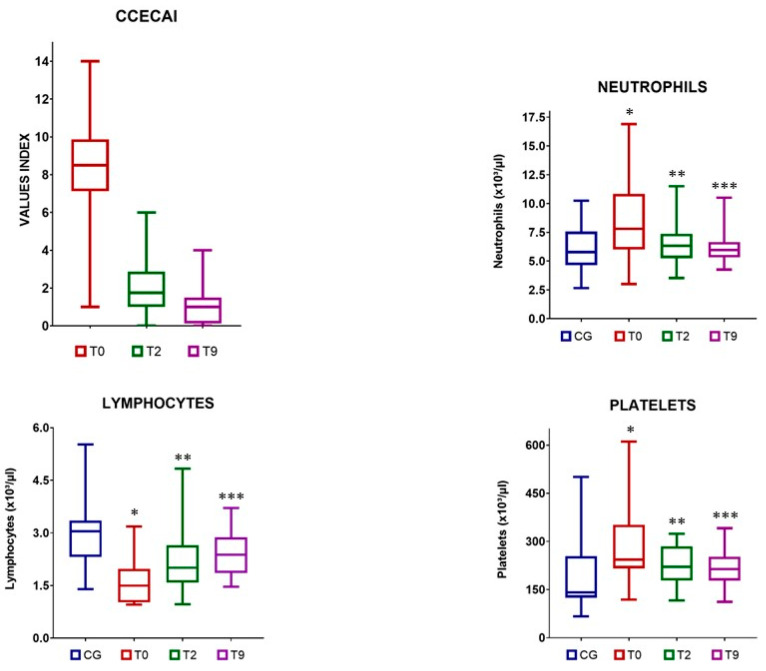
Canine Chronic Enteropathy Clinical Activity Index (CCECAI), neutrophil, lymphocyte, and platelet values in the control group (CG) and in dogs with chronic inflammatory enteropathy (CIE) treated with stem cells at different time points: before administration (T0), at 2 months (T2), and at 9 (T9) months after therapy. Neutrophils: * *p* = 0.03; effect size (95% CI): 0.78 (0.08; 1.45); ** *p* = 0.644; effect size (95% CI): −0.04 (−0.70; 0.61); *** *p* = 0.962; effect size (95% CI): −0.19 (−0.84; 0.47). Lymphocytes: * *p* < 0.0001; effect size (95% CI): −1.62 (−2.34; −0.83); ** *p* = 0.02; effect size (95% CI): −0.78 (−1.44; −0.08); *** *p* = 0.05; effect size (95% CI): −0.71 (−1.37; −0.02). Platelets: * *p* = 0.01; effect size (95% CI): 0.77 (0.07; 1.44); ** *p* = 0.063; effect size (95% CI): 0.36 (−0.31; 1.01); *** *p* = 0.171; effect size (95% CI): 0.20 (−0.46; 0.86).

The CIE group included one dog with an insignificant disease, seven dogs with moderate disease, and eight dogs with severe disease. Compared with the baseline scores, post-treatment CCECAI scores showed a significant decrease at each time point. This reduction was also significant between T2 and T9 (Table 1, Figure 1).

### 3.3. Changes in the Neutrophil, Lymphocyte, and Platelet Counts

The dogs with CIE had a significantly higher neutrophil count at T0 than the healthy dogs. After MSCs infusion, the number of neutrophils decreased significantly at T2 and T9 compared to that at T0. No differences were observed between T2 and T9 (Table 1, Figure 1). There were no significant differences between the reference values and the number of neutrophils in the dogs with CIE, both at T2 and T9 (Figure 1).

The lymphocyte number at T0 was significantly lower in the dogs with CIE than in the healthy dogs. Although the lymphocyte numbers increased after treatment, significant differences were observed only between T0 and T9 and not between T0 and T2 in the CIE dogs (Table 1, Figure 1). The lymphocyte counts in the CIE group were significantly lower than the reference values at T2; however, no such differences were observed at T9 (Figure 1).

Compared to the reference values, platelets were significantly elevated in the CIE group prior to MSCs administration. However, a significant decrease in platelet counts was observed in these dogs at T9 (Table 1, Figure 1). Furthermore, the post-treatment platelet counts were not significantly different from the reference values (Figure 1).

### 3.4. Blood Inflammatory Indices

**Figure 2:**
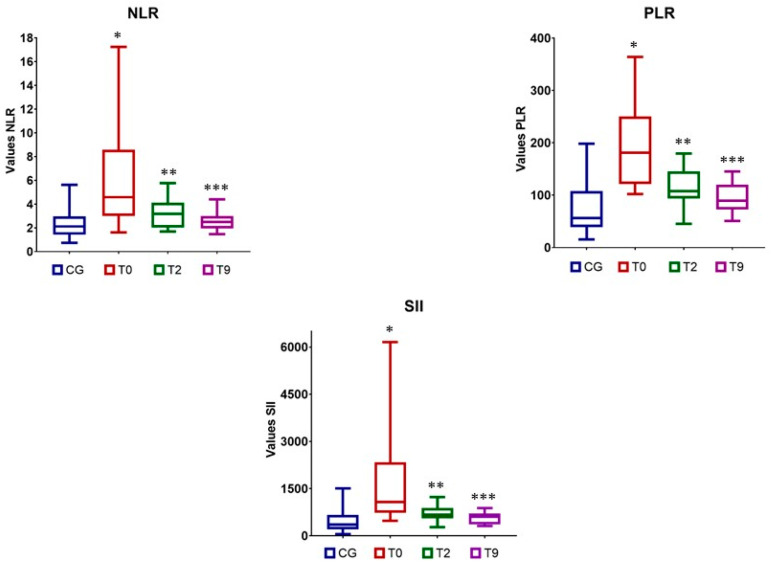
Neutrophil-to-lymphocyte ratio (NLR), platelet-to-lymphocyte ratio (PLR), and systemic immune-inflammation index (SII) in the control group (CG) and in dogs with CIE treated with mesenchymal stem cells at the different time points: before treatment (T0), at 2 months (T2), and at 9 (T9) months after therapy. NLR: * *p* < 0.0001, effect size (95% CI): 1.29 (0.54; 1.98); ** *p* = 0.02; effect size (95% CI): 0.66 (−0.03; 1.32); *** *p* = 0.298, effect size (95% CI): 0.14 (−0.52; 0.79). PLR: * *p* < 0.0001, effect size (95% CI): 1.94 (1.11; 2.68); ** *p* = 0.005; effect size (95% CI): 0.96 (0.24; 1.63); *** *p* = 0.04; effect size (95% CI):0.52 (−0.16; 1.18). SII: * *p* < 0.0001, effect size (95% CI): 1.28 (0.54; 1.97); ** *p* = 0.008; effect size (95% CI): 0.76 (0.06; 1.42); *** *p* = 0.131, effect size (95% CI): 0.27 (−0.39; 0.93).

Pretreatment NLR, PLR, and SII values of the dogs with CIE were significantly higher than those of healthy dogs. After infusion, a significant decrease was observed at T2 and T9 for all three ratios compared with the T0 pretreatment values. In addition, the difference in the drop between T2 and T9 was significant. (Table 1, Figure 2). Significant differences were observed between NLR, PLR, and SII values at T2 and the corresponding reference values. However, the NLR and SII values at T9 did not differ significantly from the reference values. The PLR of treated dogs remained significantly above the reference value at T9, but the effect size was smaller than that between T0 and the reference value.

### 3.5. Association between Inflammatory Indices and the Parameters Analyzed

**Figure 3:**
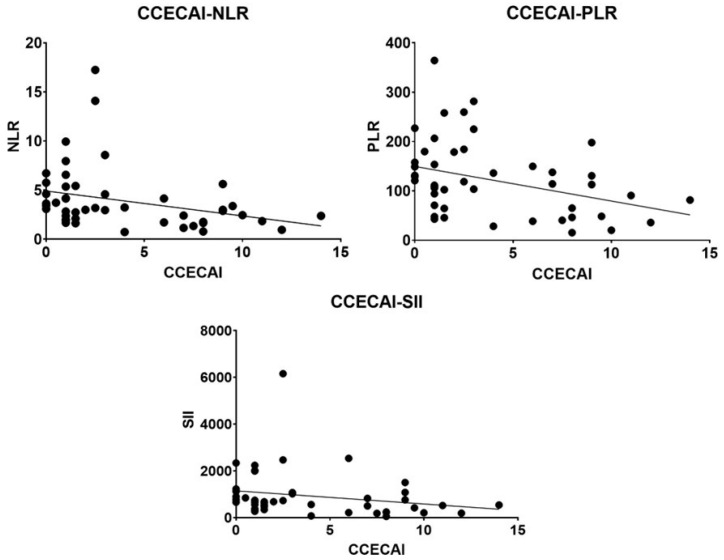
Correlation between CCECAI and each white blood cell-based inflammatory marker. CCECAI, Canine chronic enteropathy clinical activity index; NLR, neutrophil–lymphocyte ratio; PLR, platelet-to-lymphocyte ratio; SII systemic immune-inflammation index.

**Figure 4:**
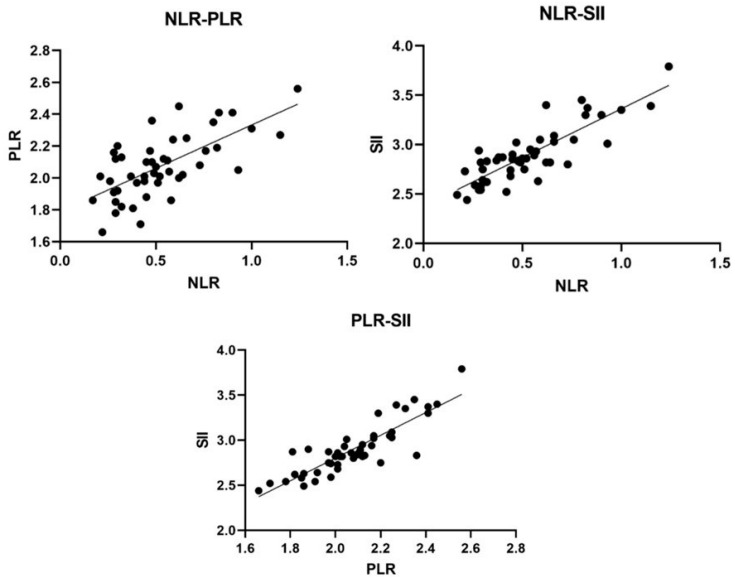
Correlation among the three inflammatory markers. NLR, neutrophil–lymphocyte ratio; PLR, platelet-to-lymphocyte ratio; SII, systemic immune-inflammation index.

A positive correlation was found between the NLR, PLR, and SII. All three blood markers were significantly associated with CCECAI and negatively correlated with lymphocyte count. Furthermore, the NLR, PLR, and SII were positively correlated with neutrophil, platelet, and platelet and neutrophil numbers, respectively (Table 2, Figure 3 and Figure 4).

## 4. Discussion

To the best of our knowledge, this is the first study to describe the values of SII in dogs and report the changes in white blood inflammatory markers over time in dogs with CIE treated with MSCs.

Our results reinforce those of earlier studies reporting that the clinical signs of dogs with CIE ameliorated after cell therapy [6,8]. The clinical improvement was accompanied by a decrease in the NLR, PLR, and SII values after treatment with Ad-MSCs.

In this study, dogs with CIE had higher neutrophil and platelet counts and lower lymphocyte counts than healthy dogs. Similar modifications associated with inflammatory processes have been described in other studies [17,27]. Reactive thrombocytosis was also reported in 6–32% of dogs with chronic enteropathy [28].

After cell therapy, we observed a decline in neutrophil and platelet counts and an increase in lymphocyte count in dogs with CIE, with these values returning to normal. A study analyzing the peripheral immune changes in patients with CD treated with autologous hematopoietic stem cell transplantation reported a transient increase in the percentage of circulating Treg cells during the first six months [29]. Systemic administration of MSCs was shown to attenuate subsequent polymorphonuclear neutrophil-predominant inflammatory responses linked to experimental ventilator-induced lung injury in rats [30].

Alterations in NLR, PLR, and SII are often considered in human CD studies. However, these biomarkers have been poorly studied in dogs with CIE.

In the present study, the NLR reference value was in the range reported by other authors [17,18,31], and our group of dogs with CIE showed an NLR value consistent with that reported for dogs with similar CCECAI [18]. However, our PLR reference value was below that described in a previous study on PLR in dogs [31], and the median PLR values of the dogs with CIE were slightly higher than the only value previously described for the same disease [19]. These discrepancies might be due to the fact that normal ranges are unknown, and researchers estimated cutoff points within their sample population. The differences in the study’s design and sample sizes might result in an inconsistent range of cutoff points [32,33,34].

The NLR was significantly higher in dogs with CIE than in healthy dogs in the present study, as previously reported in humans [35,36] and dogs [17]. PLR and SII between dogs with CIE and healthy dogs have never been compared; however, it has been demonstrated that both markers are higher in human patients with ulcerative colitis than in controls [14,15,16,37].

In the present study, NLR, PLR, and SII decreased after MSCs infusion. In dogs with CIE, the frequency of thrombocytosis was shown to reduce after immunosuppressant treatment (from 32% to 23%) [28]. In humans, the NLR of patients with active Behçet’s disease showed a significant reduction after colchicine-corticosteroid treatment; however, it remained higher than that of inactive patients or healthy controls [38]. In contrast, in this study, only the PLR values remained higher in the treated dogs than in healthy dogs.

In a study on patients with ulcerative colitis who received antitumor necrosis factor drugs, a decrease in baseline NLR and PLR was observed at week 8; this decrease was maintained till week 54 of treatment [14]. Similarly, in this study, a decrease in the inflammatory indices was observed at two months after infusion, as well as at the 9-month follow-up, where the levels remained low and within the normal range.

Moreover, this study confirms the positive correlation between NLR [17,18] and PLR [19] and disease severity, as observed by previous authors, as well as the correlation between SII and disease severity. In humans with ulcerative colitis, the decrease in NLR and PLR was correlated with clinical remission after treatment with antitumor necrosis factor drugs [14].

Our results reinforce the conclusions of earlier studies, providing further evidence that NLR can be used to determine the severity of canine CIE, suggesting that it could be used as a routine test for CIE activity and in patient monitoring [17,18], similar to that of humans.

Changes in other inflammatory markers after cell therapy have also been analyzed in dogs. Topical administration of MSCs has been shown to significantly decrease CD4, IL-1, IL-6, and tumor necrosis factor-alpha (TNF-α) levels in dogs affected by keratoconjunctivitis sicca [39].

Similarly, a decrease in CD8+ T lymphocytes, serum, and synovial C-reactive protein, was reported in dogs with ruptures of the cranial cruciate ligament at 4 and 8 weeks after intravenous and intra-articular injection of autologous canine bone marrow MSCs [40].

The mechanisms that control the dysregulated immune response following MSCs administration are still not entirely clear. The response of the MSCs to immune cells is highly complex and depends on the inflammatory signals in the microenvironment. MSCs can exert both pro- and anti-inflammatory effects; they adopt an anti-inflammatory phenotype during inflammatory conditions but maintain a proinflammatory phenotype in the absence of inflammation [12,41,42].

In tissues with a low concentration of TNF-α, interferon-gamma (IFN-γ), and other inflammatory cytokines, MSCs acquire an inflammatory phenotype after their engraftment, endorsing the host defense against infections [41,42]. Conversely, MSCs develop an anti-inflammatory phenotype when engrafted in tissues with high concentrations of the before mentioned inflammatory cytokines [42]. The increase in cytokines produced by the inflammatory immune cells benefits the generation of an immunoregulatory phenotype in MSCs and activates the further secretion of MSC-derived immunosuppressive soluble factors. Consequently, there are suppression effects of the MSCs on the immune response and inflammation boost [43,44].

The main limitation of this single-center study is the small cohort of client-owned dogs. Another limitation might be the lack of a comparison group of dogs with CIE treated with corticosteroids as standard treatment. However, the effect of corticosteroids and other immunosuppressants on white blood cells could hamper the correct interpretation of the results. It has been widely demonstrated that corticosteroids can decrease lymphocyte counts and increase peripheral granulocytes [45,46].

Furthermore, different results might be obtained according to the MSCs employed since the immunosuppressive ability of Ad-MSCs is both dose- and cell-passage-dependent [47]. Moreover, the donor age, sex, and tissue source of the MSCs might lead to significant variations in the chemical–physical characteristics of their secretome, thus affecting their immunomodulatory capacity [6,7,8,10,11,12,29].

## 5. Conclusions

This study describes the changes in white blood-cell-based inflammatory markers in dogs with CIE receiving an intravenous infusion of Ad-MSCs. Our results demonstrated that NLR, PLR, and SII decreased significantly at two months after MSCs administration, and NLR and SII reached normal levels at 9 months post-treatment. These changes in the blood inflammatory markers were accompanied by significant clinical improvement.

## Figures and Tables

**Table 1:** Descriptive statistics of the studied variables.

	Reference Value	T0	T2	T9	*p*
CCECAI		8.5 (1–14) ^a^	1.75 (0–6) ^b^	1 (0–4) ^c^	<0.0001
Effect size (95% CI)	-		2.83 (1.79; 3.73)	3.42 (2.26; 4.40)	
Neutrophils (×10^3^/µL)	5.78 (2.65–10.24)	**7.8 (3–16.9) ^a^**	6.25 (3.53–7.9) ^b^	5.88 (4.26–7.12) ^b^	0.003
Effect size (95% CI)			0.85 (0.11; 1.55)	0.96 (0.21; 1.67)	
Lymphocytes (×10^3^/µL)	3.05 (1.40–5.52)	**1.49 (0.95–3.18) ^a^**	**2 (0.96–4.83) ^a^**	2.38 (1.46–3.71) ^b^	0.039
Effect size (95% CI)			−0.76 (−1.46; −0.03)	−1.25 (−1.97; −0.46)	
Platelets (×10^3^/µL)	141 (67–501)	**243.5 (119–611) ^a^**	221 (116–324) ^a^	214 (112–341) ^b^	0.019
Effect size (95% CI)			0.60 (−0.12; 1.29)	0.76 (0.02; 1.46)	
NLR	2.11 (0.74–5.62)	**4.58 (1.62–17.24) ^a^**	**3.24 (1.68–5.76) ^b^**	2.35 (1.47–4.39) ^c^	0.002
Effect size (95% CI)			0.96 (0.20; 1.66)	1.21 (0.43; 1.93)	
PLR	56.41 (15.34–198.02)	**181.42 (102.35–364.29) ^a^**	**107.54 (45.53–179.73) ^b^**	**89.37 (50.80–145.40) ^c^**	
Effect size (95% CI)			1.28 (0.49; 2.01)	1.72 (0.87; 2.48)	
SII (×103)	360.98 (52.93–1503)	**1071.76 (475.94–6156.43) ^a^**	**662.13 (273.18–1227.55) ^b^**	547.66 (308.08–878.23) ^c^	
Effect size (95% CI)			0.99 (0.23; 1.69)	1.16 (0.39; 1.88)	

Abbreviations: CCECAI, Canine Chronic Enteropathy Clinical Activity Index; CG, control group; CIE, chronic inflammatory enteropathy; NLR, neutrophil–lymphocyte ratio; PLR, platelet-to-lymphocyte ratio; SII systemic immune-inflammation index. T0, T2, and T9, values of CIE dogs prior to treatment, 2 and 9 months after the treatment. Data are expressed as median and range (min-max). *p* values correspond to Friedman rank test. Within a row, data without a common superscript (a, b, c) differ (*p* < 0.05) according to the Wilcoxon post hoc test. Effect sizes (Cohen’s d) between T0 and the stated timepoint are given for each variable. Text in bold indicates a statistically significant difference (*p* < 0.05) with the respective normal value.

**Table 2:** Correlation study between the blood inflammatory markers and rest of parameters analyzed.

	NLR		PLR		SII	
	Spearman ρ	*p* Value	Spearman ρ	*p* Value	Spearman ρ	*p* Value
Neutrophils	0.71	<0.0001	0.38	0.01	0.73	<0.0001
Lymphocytes	−0.82	<0.0001	−0.67	<0.0001	−0.59	<0.0001
Platelets	0.003	0.99	0.54	<0.0001	0.54	<0.0001
NLR	-	-	0.62	<0.0001	0.77	<0.0001
PLR	0.63	<0.0001	-	-	0.87	<0.0001
SII	0.77	<0.0001	0.87	<0.0001	-	-
CCECAI	0.52	<0.0001	0.53	<0.0001	0.495	<0.0001

Abbreviations: CCECAI, Canine Chronic Enteropathy Clinical Activity Index; NLR, neutrophil–lymphocyte ratio; PLR, platelet-to-lymphocyte ratio; SII systemic immune-inflammation index.

## Data Availability

The data presented in this study are available on request from the corresponding author.
